# Supplementary material for: Development of an agar-plug cultivation system for bioactivity assays of actinomycete strain collections
Source: PLoS One. 2021 Nov 5;16(11):e0258934. doi: 10.1371/journal.pone.0258934 (PMC8570476; doi:10.1371/journal.pone.0258934)
Supplement: S1 File — (PDF) [file pone.0258934.s001.pdf]

Supporting Information

Development of an agar-plug cultivation system for bioactivity assays of actinomycete strain collections

Nico Ortlieb[1,2,3,#a], Elke Klenk[1,#b], Andreas Kulik[1, 4], Timo Horst Johannes Niedermeyer[1,2,3,*]

[1] Department of Microbiology/Biotechnology, Interfaculty Institute of Microbiology and Infection Medicine, Eberhard Karls University Tübingen, Auf der Morgenstelle 28, 72076 Tübingen, Germany

[2] German Centre for Infection Research (DZIF), Partner Site Tübingen, Tübingen, Germany

[3] Department of Pharmaceutical Biology/Pharmacognosy, Institute of Pharmacy, Martin-Luther-University Halle-Wittenberg, Hoher Weg 8, 06120 Halle (Saale), Germany

[4] Department of Microbial Bioactive Compounds, Interfaculty Institute of Microbiology and Infection Medicine, Eberhard Karls University Tübingen, Tübingen, Germany

[#a] Current Address: Bayer AG, Product Supply – Pharmaceuticals, Pharmaceutical Affairs, Building M231, 13353 Berlin, Germany

[#b] Current Address: Department of Microbial Interactions, IMIT/ZMBP, University of Tübingen, Auf der Morgenstelle 32, 72076 Tübingen, Germany

*corresponding author

E-mail: timo.niedermeyer@pharmazie.uni-halle.de

Taxonomy of strain Tü2700

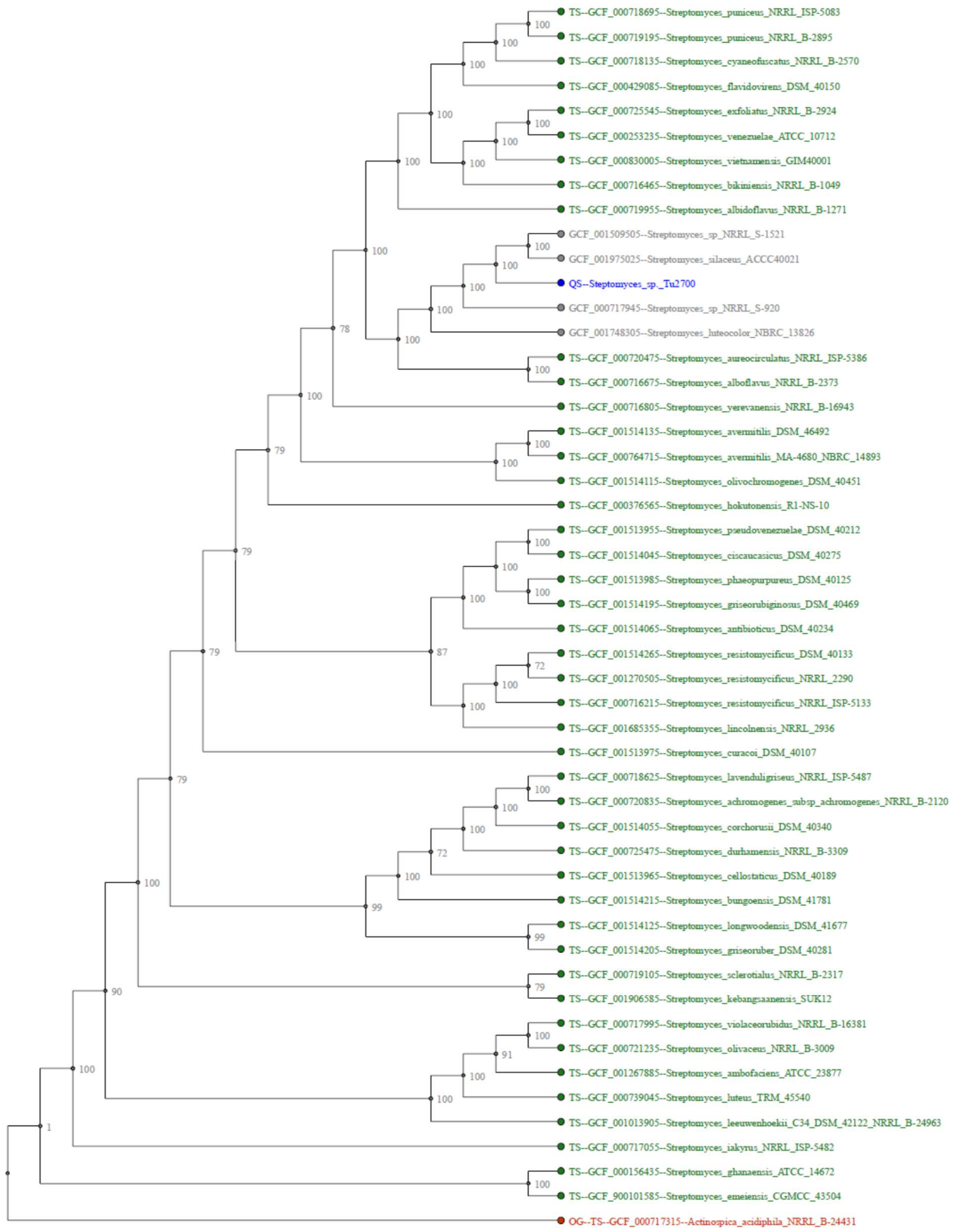

Fig. S1. Phylogenetic tree of Streptomyces Tü2700 (blue) inferred from its gDNA sequence using the software autoMLST. The phylogeny is rooted with *Actinospica acidiphila* NRRL B24431 (red), strains in green represent *Streptomyces* type strains.

Dereplication of oxazolomycin A

Previously reported UV spectra of oxazolomycin A and **1** were compared. The UV spectrum of oxazolomycin A has maxima at 265 (e, 28000), 275 (e, 34000), and 285 nm (e, 27000) due to the triene system, and one maximum at 230 nm due to the conjugated diene chromophore (Ref. 50 in manuscript). As shown in **Fig. S2**, the isolated compound exhibits UV maxima at 230 nm and 276 nm with shoulders at 265 and 285 nm. Therefore, both compounds feature the same UV absorption pattern.

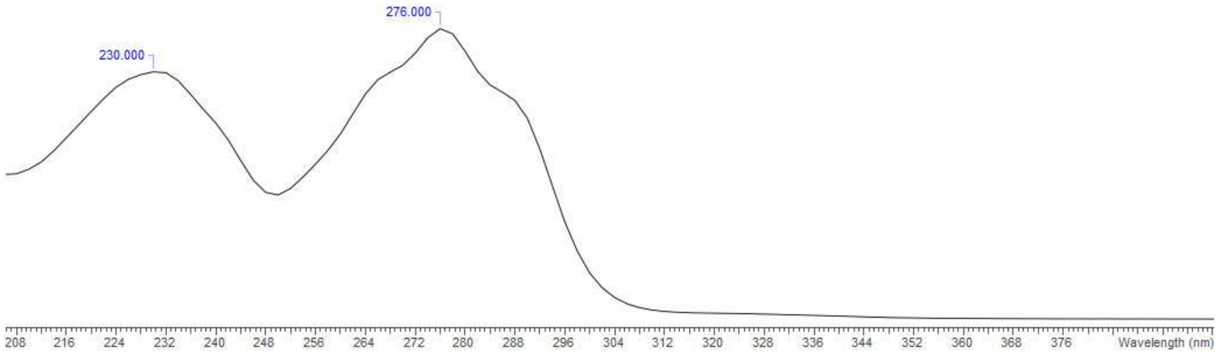

Fig. S2. UV-Spectrum of **1**, maxima at 230 nm and 276 nm with shoulders at 266 and 287 nm.

The mass of **1** was determined as 656.3 Da ($[M + H]^+$; **Fig. S3**), agreeing with the molecular mass of oxazolomycin A.

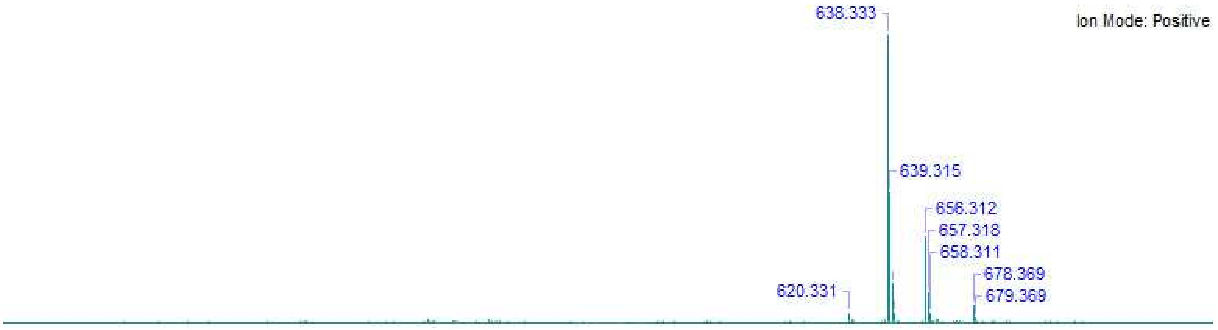

Fig. S3. Mass spectrum of **1**, pos. mode. *m/z* 638.3 ($[M + H - H_2O]^+$), *m/z* 656.3 ($[M + H]^+$), *m/z* 678.3 Da ($[M + Na]^+$).

To finally prove that **1** is identical to oxazolomycin A, **1** was dissolved in MeOH-d_4, and a ^{1}H-NMR spectrum was recorded (**Fig. S4**). Comparison of the ^{1}H NMR spectrum with literature data revealed that all observed signals were consistent with those previously described (Ref. 50 in manuscript).

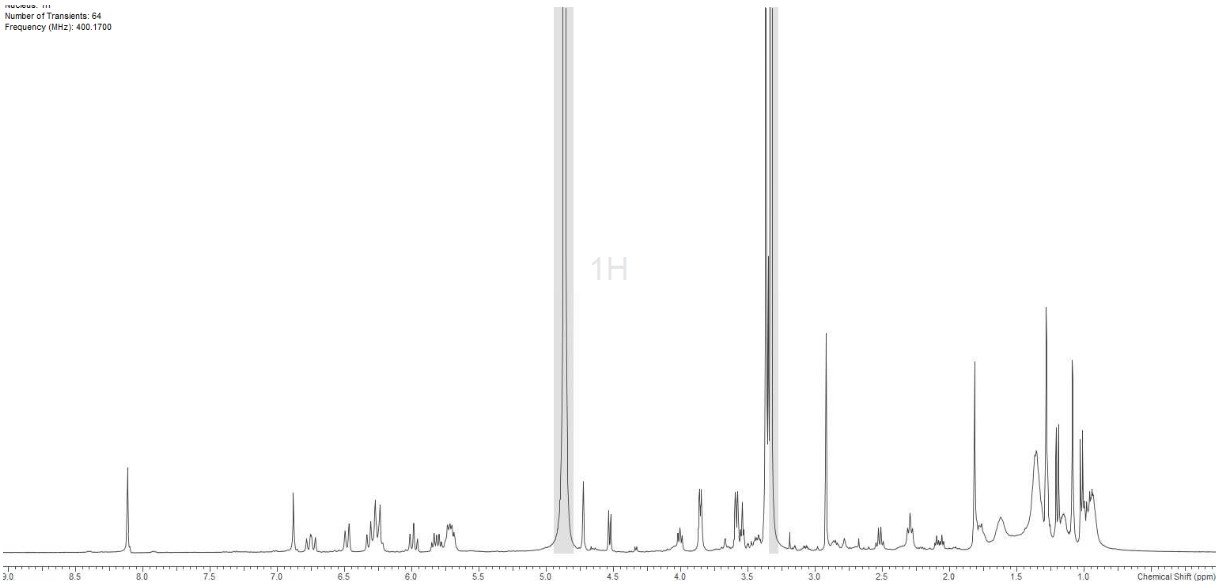

Figure S4. ^{1}H NMR spectrum of oxazolomycin A (400 MHz; methanol-d_4), shaded areas are residual solvent signals of methanol-d_4

Therefore, **1** was confirmed to be indeed oxazolomycin A.

Oxazolomycin A is also produced by the strain when grown on agar (peak at t_r 9.6 min in **Fig. S5**, UV- and MS spectra in **Figs. S6** and **S7**).

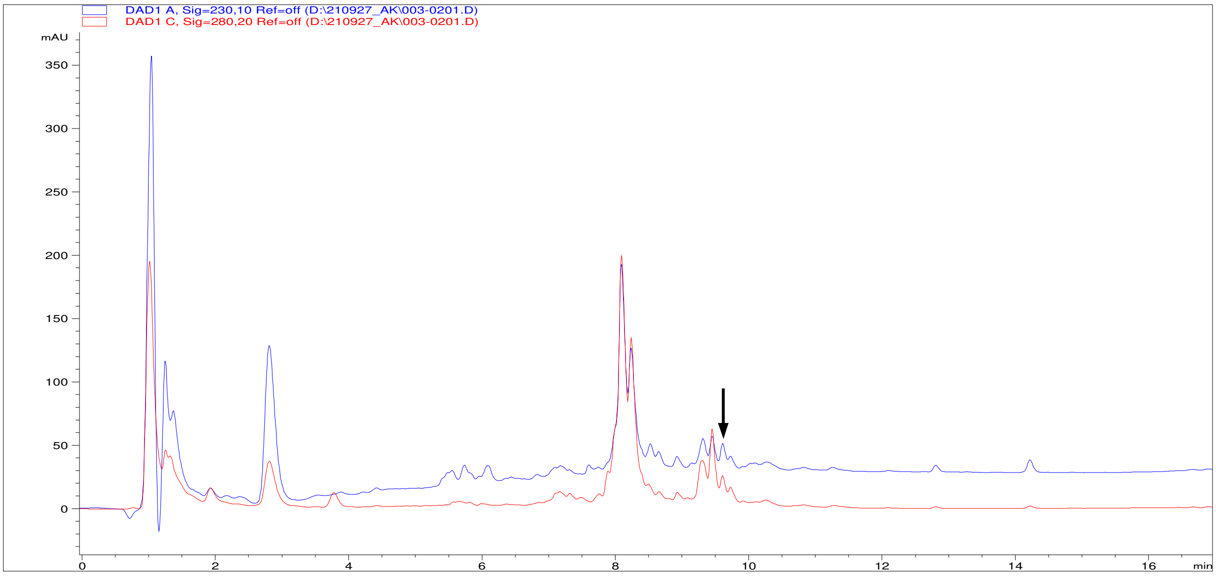

Figure S5. Chromatogram of the agar-plug extract of strain Tü2700. Peak at t_r 9.6 min (arrow) is oxazolomycin (**1**).

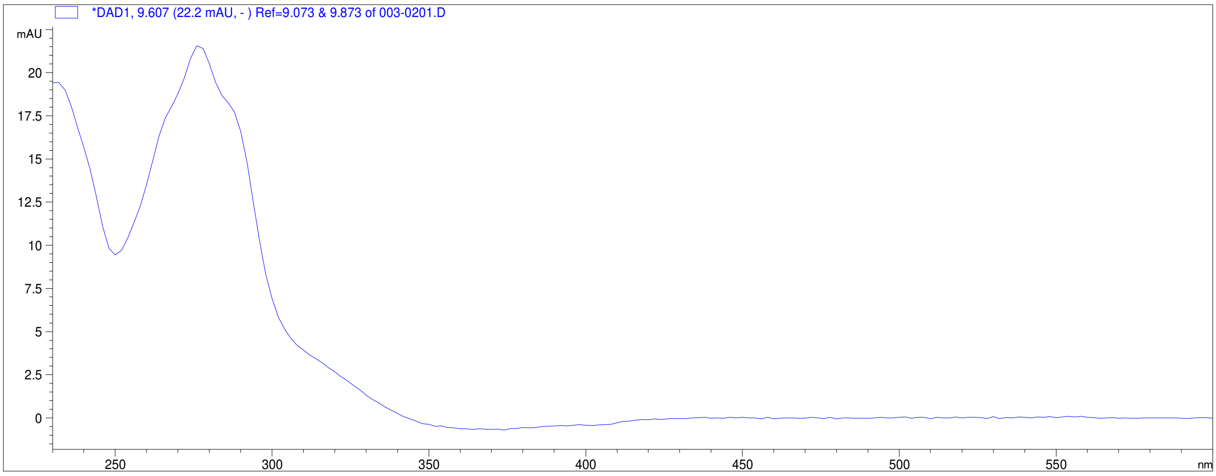

Figure S6. UV-Spectrum of the compound at t_r 9.6 min corresponds to the UV spectrum of **1** isolated from liquid cultivation medium (see Fig. S2).

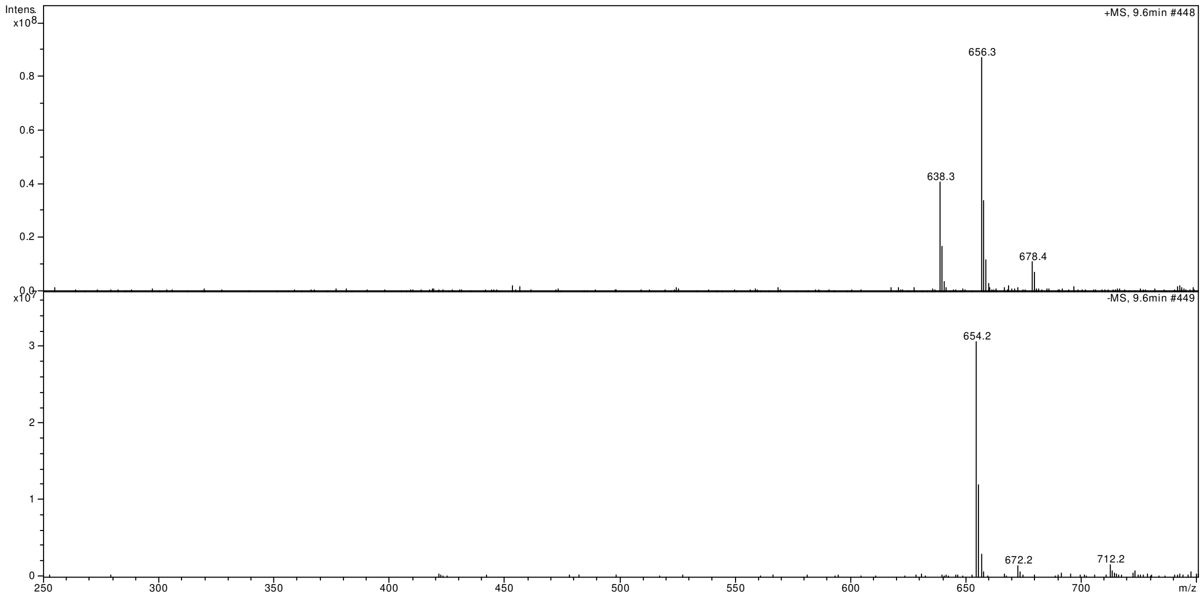

Figure S7. Mass spectra of the compound at t_r 9.6 min corresponds to the mass spectrum of **1** isolated from liquid cultivation medium (pos. mode top, neg. mode bottom; see Fig. S3).
