# Supplementary material for: Development of an agar-plug cultivation system for bioactivity assays of actinomycete strain collections
Source: PLoS One. 2021 Nov 5;16(11):e0258934. doi: 10.1371/journal.pone.0258934 (PMC8570476; doi:10.1371/journal.pone.0258934)
Supplement: Attachment — Submitted filename: 210928 _ Response to Reviewers.pdf [file pone.0258934.s002.pdf]

PONE-D-21-25646

**Rebuttal letter**

We thank the editor and the reviewers for their helpful comments and suggestions, which we have taken into account as follows:

Editor comments

***1. Please ensure that your manuscript meets PLOS ONE's style requirements, including those for file naming.***

We have double-checked to meet all formatting requirements.

***2. Please ensure that you refer to Figure 2 in your text as, if accepted, production will need this reference to link the reader to the figure.***

We now refer to Fig. 2 in the text.

***3. We note that Figure 1 in your submission contain copyrighted images.***

We are not sure how you came to this conclusion. Figure 1 does not contain copyrighted images. All the subfigures have been photographed by Nico Ortlieb, first author of this study.

***4. Please review your reference list to ensure that it is complete and correct.***

We have double-checked all the references, the list is complete and correct.

***Reviewer #1***

***1. The abstract part should be optimized. The results of this study should be described. In addition, the authors should also describe briefly what measures the authors take to address the challenges encountered in the workflow.***

The abstract has been revised. We think we already describe the results of the paper in sufficient detail (“We developed a medium-throughput cultivation and agar-plug assay workflow that allows the convenient inoculation of solid agar plugs with actinomycete spore suspensions from a strain collection, and the transfer of the agar plugs to petri dishes to conduct agar-plug bioactivity assays.” – as this is a methodology paper, this is the main result of our report…). We now describe some of the challenges.

***2. Line 85-86, the sentence “a plug---out of the agar” should be rephrased.***

The sentence has been rephrased and now reads “After a defined incubation time, a plug of the agar including the strain is punched out aseptically using e.g. a cork borer, the back of a pipet tip, or a trimmed syringe.”

***3. Line 130, the strain name S. aureus should be provided in full name for the first time.***

Done. We also have shortened Staphylococcus to S. in other places.

***4. Line 135, OD what is the wavelength?***

Wavelength is now given ($OD_{600}$).

***5. The result part “Screening of the strain collection”, how many strains were used here? The number should be provided.***

We had already given information about for the number of formatted strains, but for the sake of clarity, we have added the information here, as well.

***Reviewer #2:***
***In my opinion the greatest outstanding issue of this paper is in the identification of oxazolomycin A as the bioactive compound. Whilst oxazolomycin A is clearly the compound they have managed to isolate from Tü2700 this is following a series of liquid culture optimisations for isolation and structural elucidation. The link to it being the bioactive compound identified in the screen is unsubstantiated. This doesn't actually matter per se however I would recommend the authors make this distinction clearer and that the screen only allows for the identification of strains with the propensity to produce bioactive compounds but what is subsequently identified in the liquid isolation is most likely different to that showing activity on the agar plug screen. If the authors wished to confirm Tü2700 was producing oxazolomycin A during the screen it would be prudent to isolate the compound directly from the separate agar plug plates which, whilst it may take a significant number of plates, has been successfully achieved for years.***

This is very true. Indeed, in another screening (not discussed here), we found different antibiotic compounds being produced on solid and in liquid medium. For the presented strain, this does not seem to be the case: We have found Oxazolomycin A also in the agar plug (HPLC-MS), and the halo around samples of the liquid medium look the same hazy way as the agar plugs. Thus, we think that the compound produced on agar and in liquid medium is the same. We have explained this in more detail in the manuscript and present additional data (HPLC-MS of an extracted agar plug) in the SI.

***- The authors make the claims (e.g. line 25, 72) that the solid cultivation of actinomycetes is rarely used for bioactivity screenings. Whilst this may be true for extremely large scale screens I would argue that the classical overlay bioassay is a widespread and is indeed first line methodology for screening the bioactivity of new isolates due to its simplicity and replicability. I would recommend the authors to readjust their claims slightly.***

The claims have been adjusted, and the sentences have been written more clearly.

***- I believe the manuscript would benefit from a small explanation of inhibitors of bacterial quorum sensing, perhaps just a few sentences, to give the readers a better understanding of why they are a promising source of therapeutics.***

We have added some information about this in the introduction.

***- I believe a Figure containing images of the strain Tü2700 and its bioactivity as noted from the screen would be of great benefit to the readers.***

Thank you for this suggestion. Figure 2 has been extended to include images of the activity testing.

***o Line 25: I think it would be more correct to use "submersed" rather than "submerse" and this is continued throughout the manuscript; Line 107: Please be consistent in the use of ml or mL; Line 151: Similarly with v/v or V/V***

All "submerse" have been replaced by "submersed". "mL" and "v/v" are now consistently used.
